# Cardiac Autonomic Effects of Secondhand Exposure to Nicotine from Electronic Cigarettes: An Exploratory Study

**DOI:** 10.1097/EE9.0000000000000033

**Published:** 2019-02-12

**Authors:** Mi-Sun Lee, Vaughan W. Rees, Petros Koutrakis, Jack M. Wolfson, Youn-Suk Son, Joy Lawrence, David C. Christiani

**Affiliations:** a Environmental and Occupational Medicine and Epidemiology Program, Department of Environmental Health, Harvard T.H. Chan School of Public Health, Boston, Massachusetts; b Department of Social and Behavioral Sciences, Harvard T.H. Chan School of Public Health, Boston, Massachusetts; c Exposure, Epidemiology and Risk Program, Department of Environmental Health, Harvard T. H. Chan School of Public Health, Boston, Massachusetts; d Department of Environmental Engineering, Environmental and Marine Sciences and Technology, Pukyong National University, Busan, South Korea; e Massachusetts General Hospital/Harvard Medical School, Boston, Massachusetts.

**Keywords:** E-cigarette, Nicotine, Heart rate variability, Cardiovascular risk

## Abstract

The rapid increase in prevalence of e-cigarette (EC) use may lead to widespread exposure to secondhand emissions among nonsmokers, but evidence on the potential cardiovascular health risks is limited. We aimed to investigate the effect of short-term secondhand exposure to nicotine from e-cigarette (EC) emissions on cardiac autonomic function using heart rate variability (HRV).

**Methods:**

A randomized, repeated measures crossover study of healthy nonsmoking volunteers was conducted. Standard deviation of NN intervals (SDNN), average of the standard deviation of NN intervals (ASDNN), root mean square of successive differences (rMSSD), and heart rate–corrected QT interval (QTc) were calculated during 1 hour of EC exposure session.

**Results:**

Nicotine from EC emissions was associated with a 7.8% decrease in SDNN (95% CI [confidence interval]: −11.2%, −4.3%), 7.7% decrease in ASDNN (95% CI: −11.0%, −4.2%), and 3.8 milliseconds decrease in QTc (95% CI: −5.8, −1.9). Compared with a short exposure time period (<15 minutes), greater nicotine associated with reductions in ASDNN (*P*_for interaction_ = 0.076) with longer exposure time periods. For QTc, greater nicotine associated with reductions were found during 15- to 30-minute exposure time period (*P*_for interaction_ = 0.04).

**Conclusion:**

We present the first evidence of cardiac autonomic effects of short-term secondhand exposure to nicotine from EC emissions among healthy nonsmokers. Further comprehensive research on EC exposure extending to more subjects and flavor compounds is warranted.

## Abstract 2

Supplemental Digital Content is available in the text.

**What this study adds**

The rapid increase in the use of electronic cigarette (EC) may lead to widespread exposure to secondhand emissions among nonsmokers, but evidence on potential cardiovascular effects of EC exposure is limited. To our knowledge, this is the first study addressing the effect of short-term exposure to nicotine from EC emissions on cardiac autonomic function in general healthy nonsmokers. Greater effect was observed during longer exposure to EC emissions, implying that nicotine from EC emissions may have both acute and cumulative cardiac effects. Further studies are needed to confirm our findings.

## Introduction

The prevalence of use of electronic cigarettes (hereafter “EC”) has increased rapidly since their introduction to the United States in 2007.^1^ In 2014, some 48% of current smokers and 55% of former smokers in the United States tried an EC.^2^ Uses among US teens have increased at an alarming rate; eight-fold increase in the years from 2011 to 2016, from 1.5% to 11.3% among high school students.^3^ The emissions of ECs contain a mixture of chemicals including mainly of nicotine, propylene glycol, glycerin, flavors, and other additives, which are present in the particulate and gaseous forms.^4–6^ While concerns regarding the toxicity of EC emissions focus primarily on additives and fine particles, there is increasing recognition of the impact of nicotine. Nicotine can induce elevation of blood pressure and heart rate and deregulation of cardiac autonomic function, mainly indicated by heart rate variability (HRV), via activation of the sympathetic nervous system with release of norepinephrine and epinephrine.^7^ However, to date, there has been no study of the cardiac autonomic effects of nicotine arising from passive exposure to EC emissions. We investigated the effects of short-term secondhand exposure to nicotine from EC emissions on cardiac autonomic function among healthy, nonsmoking adults.

## Methods

### Study design and subjects

The study was approved by the Institutional Review Boards of the Harvard T.H. Chan School of Public Health (Protocol no. 14–2108). All subjects gave written informed consent before participating in the study. Our study is a randomized repeated measures crossover study that builds on the Harvard-National Institute of Environmental Health Science Center for Environmental Health pilot study (Grant no. P30ES000002). Participants were five healthy nonsmoking adults (fewer than 100 cigarettes in lifetime and no smoking in the past 30 days) without cardiovascular disease and with no current use of any medication recruited from the Harvard T.H. Chan School of Public Health in Boston, MA, during March to May 2015. Each participant completed a modified American Thoracic Society (ATS) questionnaire which also included information on sociodemographic factors including age and gender. Body mass index (BMI, kg/m^2^) was calculated by weight in kilograms divided by the square of height in meters. Two EC exposure sessions were each conducted over two consecutive days, with sessions lasting for 1 hour.

### Analysis of nicotine from electronic cigarette emission

A detailed description for the EC used and analysis of nicotine emitted from EC has been provided previously.^6^ Briefly, an EC containing 1.8% nicotine, a popular US brand, was used for each EC exposure session. We used an automatic multiple smoking machine (Modified TE-2 system, Teaque Enterprises, Davis, CA) to provide two standard puffs per minutes. Twenty-five percent of the flow from the smoking machine was diluted using particle free, humidified room air in a mixing tube at an output flow of 120 LPM into a cone, from which the participant breathed the diluted EC vapor in a sitting position with breathing as usual. Dilution ratio (1:370) was calculated to be approximately equivalent to that of an exposure chamber (27 m^3^) with an air exchange rate of 1 per hour. All subjects were blinded to which EC brand used and levels of nicotine. Nicotine concentrations from EC emissions were measured using Gas Chromatography(GC)/Tandem Mass Spectrometry(MS/MS) (Enthalpy Analytical, Inc., Durham, NC) following active sampling on XAD-7 sorption tubes (SKC, Inc., Valley View Road Eight Four, PA) at a flow rate of 1 L/minute for 60 minutes during EC exposure session.

### Heart rate variability and corrected QT interval measurements

We used HRV and heart rate–corrected QT (QTc) interval as a measure of cardiac autonomic response to nicotine exposure from EC emission. We followed the protocols for subject preparation and the ECG monitoring similarly used by our previous epidemiologic studies.^8,9^ The electrocardiogram (ECG) of each individual was monitored continuously using a five-lead ECG Holter monitor, a DigiTrak XT Holter Recorder (Philips Medical Systems, Andover, MA). The Holter monitor was calibrated 15 minutes before placing electrodes. Separate electrodes were placed on the participant’s skin, and if necessary, the area was shaved for proper adhesion, and the leads were periodically checked by study staff. Each recording was sent to First Call Medical, Inc. (Billerica, MA) for processing and analysis using Philips Zymed Holter 2010 Plus software and then screened to correct data artifacts. A trained professional, blinded to exposure condition, performed all analyses and edited all normal and abnormal findings based on standard procedures. The mean of SD of normal-to-normal intervals (SDNN, in milliseconds), average of the standard deviation of NN intervals (ASDNN, in milliseconds), root mean square of successive differences (rMSSD, in milliseconds), QTc (in milliseconds), and the mean heart rate (HR, in beats per minute) were calculated in 15-minute segments during 1-hour exposure session.

### Statistical analysis

Data were analyzed using PROC MIXED in the SAS statistical package version 9.4 (SAS Institute Inc, Cary, NC). We treated health outcome variables (SDNN, ASDNN, rMSSD, and QTc) as repeated measurements in 15-minute segments and SDNN, ASDNN, and rMSSD were log_10_-transformed to improve normality and stabilize the variance. Linear mixed effects models with random intercepts and unstructured covariance were used to estimate the percent changes as (10*^β^* − 1) × 100%, with 95% CI [10^(*β*±1.96×SE)^ − 1] × 100%, where *β* and SE are the estimated regression coefficient and its standard error, in 15-minute segments for 1 µg/m^3^ increase in nicotine from EC aerosols. We compared the model fit using the −2 log likelihood (2-LogL), Akaike Information Criterion (AIC), and the Bayesian Information Criterion (BIC; eTable 1; http://links.lww.com/EE/A26). Finally, we choose age- and BMI-adjusted models (M_5_) and BMI-adjusted model (M_6_) as main models which present lower 2-LogL, AIC, and BIC values, indicating a better fit. To assess effect modification by exposure time periods (<15, 15–30, 30–45, 45–60 minutes), multiplicative interaction terms along with the main effects were included in the models. Results are given as estimated percent changes with their 95% confidence intervals (CIs) in 15-minute HRV per 1 µg/m^3^ increase in nicotine from EC emissions.

## Results

Participant mean (SD) age was 29.4 (6.0) years and 40% were female. Mean BMI (SD) was 22.8 (1.9) kg/m^2^. Mean (SD) nicotine concentration was 4.8 (2.3) µg/m^3^ (Table 1).

**Table 1:**
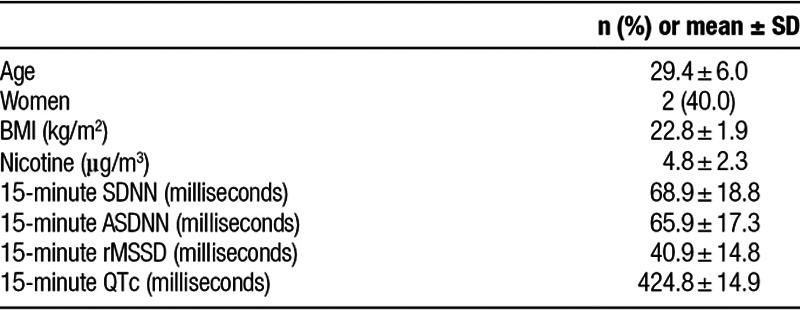
Characteristics of study participants

Table 2 shows the associations of 15-minute HRV and 15-minute QTc with nicotine concentrations during 1-hour exposure to EC emissions. We found that nicotine concentration during 1-hour exposure to EC emissions was significantly associated with decreases of 7.8% (95% CI: −11.2%, −4.3%) in 15-minute SDNN, 7.7% (95% CI: −11.0%, −4.2%) in 15-minute ASDNN, and 3.8 milliseconds (95% CI: −5.8, −1.9) in 15-minute QTc after adjusting for potential covariates such as age and BMI (eTable 2; http://links.lww.com/EE/A27).

**Table 2:**
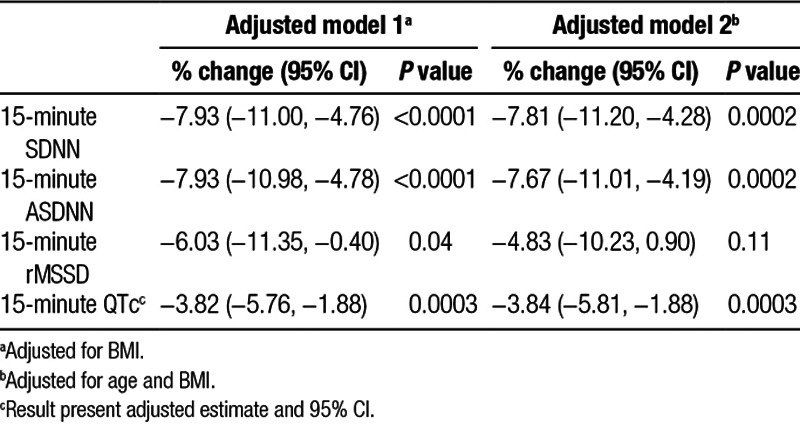
Adjusted percent changes (95% CIs) in HRV and QTc interval associated with nicotine (µg/m^3^) in EC emission

We also assessed effect modification by exposure time period in the association between secondhand exposure to EC emission, measured by nicotine concentrations, and outcomes. Compared with a short exposure time period (<15 minutes), greater nicotine associated with reductions in ASDNN (*P*_for interaction_ = 0.076) with longer exposure time periods. For QTc, greater nicotine associated with reductions were found during 15- to 30-minute exposure time period (*P*_for interaction_ = 0.04; Figure).

**Figure.**
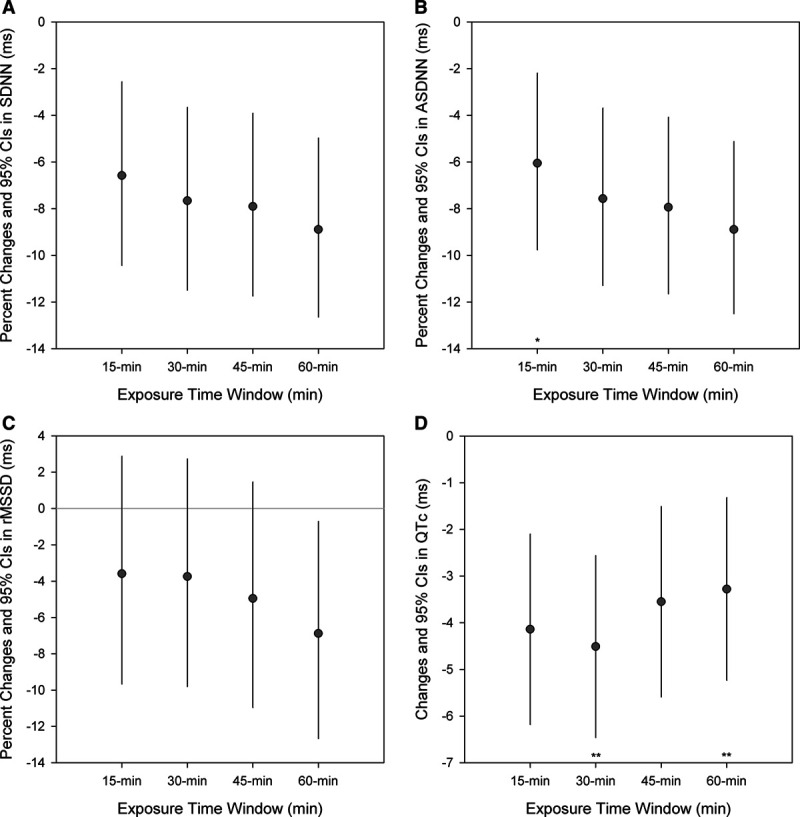
Adjusted percent changes and 95% CIs in HRV and QTc interval per 1 µg/m^3^ increase in nicotine concentration in EC emission by exposure time windows (<15, 15–30, 30–45, and 45–60 minutes). Models were adjusted for age and BMI. Circle symbols indicate the effect estimate. *P_for interaction_ < 0.1, **P_for interaction_ < 0.05.

## Discussion

We found an association between short-term secondhand exposures to EC emissions, measured by nicotine concentrations, and decreased HRV as well as shortening of the QTc, both markers of cardiovascular risk, in healthy nonsmoking adults. More pronounced declines in HRV were found during longer exposure to EC emissions, implying that nicotine exposure may have both acute and cumulative cardiac effects.

Although EC emissions are devoid of the by-products of tobacco combustion, they are nonetheless, a source of nicotine.^10^ Nicotine exposure via cigarette smoking is known to alter cardiovascular autonomic function,^11^ and nicotine exposure from noncombusted sources, such as oral nicotine products, has shown a significant reduction in HRV in healthy nonsmoking adults.^12^ In a cross-sectional case–control study, habitual EC use was associated with a shift in cardiac autonomic balance toward sympathetic predominance following acute exposure to EC with nicotine (1.2%) within 30 minutes of exposure.^13^ The decrease in high-frequency (HF) and an increase in the low-frequency (LF) component were significantly greater with exposure to EC with nicotine compared with the EC without nicotine.^14^ Elevated heart rate and plasma nicotine were also reported after 5 minutes of the first puff, and throughout 1 hour of the ad-lib period in EC users.^15^ In a recent study of the National Health Interview Surveys of 2014 and 2016, daily EC use, adjusted for other risk factors including smoking conventional cigarette, is associated with increased risk of myocardial infarction (Odds ratio: 1.79, 95% CI: 1.20, 2.66; *P* = 0.004).^16^

The levels of nicotine in the present study (mean: 4.8 µg/m^3^) was slightly higher than those in secondhand EC exposure in public EC conventions and events (median: 1.1 µg/m^3^)^17^ and those in secondhand exposure to EC emissions (mean: 2.51 µg/m^3^, ranged from 0.82 to 6.23 µg/m^3^) but lower than those in cigarettes smoking in the chamber study,^18^ suggesting that exposure level in our study is comparable to the secondhand EC exposure. Animal study found that mice exposed to EC vapor containing nicotine showed impaired lung growth,^19^ but to our knowledge, human health effects of passive exposure to EC has not yet been well studied.

Although direct comparison between cigarette smoking and EC is limited, both contain multiple chemicals, which may have potential health concerns.^20^ A body of evidence has published to date on the effects of smoking, both active and passive, on decreased HRV and their association with adverse cardiovascular health consequences.^21^ Two main mechanistic pathways, the nicotinic pathway and fine particles toxicity, have been proposed to explain the adverse effects of smoking on cardiac autonomic function. Nicotine, the main constituent of tobacco smoke, can affect cardiac autonomic function through neurohormonal regulation of the circulatory system, characterized by increased sympathetic activity and reduced parasympathetic activity.^7^ Nicotine modulates the autonomic nervous system (ANS) by activating and desensitizing nicotinic acetylcholine receptors (nAChRs), which are pentameric ligand-gated ion channels superfamily widely expressed in peripheral and central nervous system, autonomic ganglia, neuromuscular junctions, and non-neuronal tissues.^22^ Stimulation of nAChRs by nicotine results in the release of neurotransmitters such as acetylcholine (ACh), dopamine, and norepinephrine^23^ thereby affecting heart rate.^24^ Experimental research using rat hearts suggests that nAChRs in ganglia were found to be involved in the autonomic regulation of cardiovascular activities.^25^ Plasma catecholamine levels increased within 1 minute after smoking a cigarette.^21^ Particles from the incomplete combustion of cigarette smoking also have known to play an important role in the smoking-induced reduction in HRV. Particles could stimulate afferent nerves in the lungs which influence the autonomic nervous system. In a study of healthy volunteers, inhaled particles rapidly pass into the systemic circulation within minutes: detected in blood at 1 minute, reached a maximum concentration between 10 and 20 minutes.^26^ We previously reported that fine and nanoparticles are present in EC emission.^6^ The median concentration of PM_2.5_ was 21.1 µg/m^3^, which was similar to the airborne concentrations in indoor and outdoor assessments of passive exposure^27,28^ and passive exposure to EC emissions in a simulated café^29^ and exceeded the annual mean of National Ambient Air Quality Standards (NAAQS, 12 µg/m^3^) established by the US Environmental Protection Agency (EPA) and WHO guidelines (10 µg/m^3^). PM_2.5_ exposure from SHS was associated with decreased HRV.^30^

We found that 1 µ/m^3^ nicotine was associated with the declines of 7.8% in SDNN, 7.7% in ASDNN, and 3.8 milliseconds in QTc (Table 2). In a study among 14 restaurant or bar workers, 1 mg·hour/m^3^ PM_2.5_ by ETS exposure was associated with a decrease of 2.7% in SDNN and 3.8% in rMSSD.^31^ In a study of 35 boilermaker workers who were exposed to ETS relatively long-hours, for around 6 hours, greater effects of ETS exposure on HRV were observed: 7.5% decrease in rMSSD and 14.7% decrease in high-frequency (HF) power associated with the 15-minute PM_2.5_ moving averages. When they assessed with longer exposure (4-hour moving average of PM_2.5_), greater decrease of 46.9% in rMSSD and 77.7% in HF power were found.^32^ Although the magnitude of reduction in HRV from previous studies is difficult to compare quantitatively with because of differences in study designs, exposure measurement, and assessment of HRV, these studies showed that HRV reduction was associated with the ETS exposure.

We acknowledge several limitations. First, this exploratory study is limited by the small number of subjects, limiting generalizability. The use of a repeated measure design, however, provided power to detect the effects. Second, the possibility that other EC ingredients besides nicotine (e.g., flavorants) may confound the effects we observed but using the single flavor EC prevents controlling the potential effect of flavor. Third, we did not measure plasma nicotine levels, a biomarker of nicotine exposure. To confirm and support our findings and to give more insight into cardiovascular health implication, further epidemiologic studies are needed to investigate the association between nicotine exposure via secondhand EC smoking in the general environment, biomarker of nicotine exposure, and change in cardiac autonomic function and disease. Finally, nicotine and constituents delivered via machine-generated EC emissions and vapor exhaled by human may differ although nicotine levels in EC emission via smoking machine generation was found to similar to those of exhaled from EC user.^17^ Given these limitations, our findings warrant confirmation in future studies.

## Conclusion

Our findings suggest cardiac autonomic effects of short-term secondhand exposure to nicotine from EC emissions in healthy nonsmokers. Further research with larger samples, involving EC emissions with varying levels of exposure to nicotine and flavor compounds are needed to more fully understand the cardiac autonomic toxicity of nicotine delivered via EC emission. These finding may also guide further consideration of EC regulations, including broader adoption of state and local laws intended to protect the public from exposure to EC emissions and proposed Food and Drug Administration health warnings.

## Conflicts of interest statement

The authors declare that they have no conflicts of interest with regard to the content of this report.

## Acknowledgments

This publication was made possible by US EPA Grant Number RD-834798 and National Institute of Environmental Health Science (NIEHS) Grant Number P30ES000002. Its contents are solely the responsibility of the grantee and do not necessarily represent the official views of the US EPA or the NIEHS. Further, US EPA and NIEHS do not endorse the purchase of any commercial products or services mentioned in the publication. The authors would like to sincerely thank all participants and Li Su for help in data collection in this project.

## Supplementary Material
