# Supplementary material for: Cardiac Autonomic Effects of Secondhand Exposure to Nicotine from Electronic Cigarettes: An Exploratory Study
Source: Environ Epidemiol. 2019 Feb 12;3(1):e033. doi: 10.1097/EE9.0000000000000033 (PMC6814191; doi:10.1097/EE9.0000000000000033)
Supplement: Supplementary file 1 [file ee9-3-e033-s001.pdf]

Supplementary table. Results of AIC and BIC analysis for 6 competing models

| Model | -2logL | AIC | BIC |
|---|---|---|---|
| $M_1$: adjusted for session | -78.8 | -64.8 | -67.5 |
| $M_2$: adjusted for gender | -78.3 | -68.3 | -70.2 |
| $M_3$: adjusted for age | -78.6 | -68.6 | -70.6 |
| $M_4$: Base model (unadjusted) | -78.3 | -70.3 | -71.9 |
| $M_5$: adjusted for age and BMI | **-82.9** | **-70.9** | **-73.3** |
| $M_6$: adjusted for BMI | **-82.9** | **-72.9** | **-74.9** |

*Note*. Result in bold indicate the better fitting model
