# Supplementary material for: Cardiac Autonomic Effects of Secondhand Exposure to Nicotine from Electronic Cigarettes: An Exploratory Study
Source: Environ Epidemiol. 2019 Feb 12;3(1):e033. doi: 10.1097/EE9.0000000000000033 (PMC6814191; doi:10.1097/EE9.0000000000000033)
Supplement: Supplementary file 2 [file ee9-3-e033-s002.pdf]

Supplementary table 2. Characteristics of the study subjects (n = 5)

Subject	Age	Gender	Visit	BMI	Nicotine	ASDNN	SDNN	RMSSD	QTc	Exposure Time (min)
1	41	M	1	23.36	7.08	68.1	67.2	26.6	421	<15
1	41	M	1	23.36	7.08	48.9	47.1	26.6	413	15-30
1	41	M	1	23.36	7.08	60.6	80.2	58.9	418	30-45
1	41	M	1	23.36	7.08	56	59.4	52	413	45-60
1	41	M	2	23.36	5.39	42.4	42.4	21.1	432	<15
1	41	M	2	23.36	5.39	47.6	52.9	24.1	432	15-30
1	41	M	2	23.36	5.39	52.4	53.8	24.2	431	30-45
1	41	M	2	23.36	5.39	50.6	52.8	22	433	45-60
2	26	F	1	21.64	8.51	62.7	64.7	68.1	415	<15
2	26	F	1	21.64	8.51	50.2	55.8	50.6	419	15-30
2	26	F	1	21.64	8.51	47.1	47.8	34.2	434	30-45
2	26	F	1	21.64	8.51	34.1	39.4	27.2	446	45-60
2	26	F	2	21.64	4.99	74.8	78.4	57.8	445	<15
2	26	F	2	21.64	4.99	65.2	63.3	61.2	429	15-30
2	26	F	2	21.64	4.99	49.9	48.2	46.2	443	30-45
2	26	F	2	21.64	4.99	49.3	46.9	36.6	443	45-60
3	28	M	1	20.20	5.69	77.1	81.8	42.3	404	<15
3	28	M	1	20.20	5.69	83	84.7	59.5	398	15-30
3	28	M	1	20.20	5.69	82.2	79.2	34.3	404	30-45
3	28	M	1	20.20	5.69	73.1	82.8	33.4	401	45-60
3	28	M	2	20.20	7.06	78.3	81.2	42.7	399	<15
3	28	M	2	20.20	7.06	82.8	83.5	40.2	398	15-30
3	28	M	2	20.20	7.06	65.1	65.1	31.8	396	30-45
3	28	M	2	20.20	7.06	67.7	64.8	26.1	400	45-60
4	27	F	1	22.94	2.33	68.2	66	46.9	423	<15
4	27	F	1	22.94	2.33	53.8	58.8	34.1	430	15-30
4	27	F	1	22.94	2.33	59.3	65.9	36.9	437	30-45
4	27	F	1	22.94	2.33	83.6	86.9	50.3	429	45-60
4	27	F	2	22.94	1.36	81.1	79.3	56	433	<15
4	27	F	2	22.94	1.36	101	113.9	70.3	435	15-30
4	27	F	2	22.94	1.36	127.4	128.8	83.5	430	30-45
4	27	F	2	22.94	1.36	91	110	52.4	438	45-60
5	25	M	1	25.80	2.86	66.8	64.5	31.7	426	<15
5	25	M	1	25.80	2.86	62.8	70.7	34.8	437	15-30
5	25	M	1	25.80	2.86	61.7	67.5	34.2	436	30-45
5	25	M	1	25.80	2.86	64.3	63.9	34	434	45-60
5	25	M	2	25.80	3.06	65.9	67.9	32.8	433	<15
5	25	M	2	25.80	3.06	58.1	62.6	30.4	432	15-30
5	25	M	2	25.80	3.06	57	60.5	30.1	435	30-45
5	25	M	2	25.80	3.06	63.6	64	29.3	438	45-60
